# Dimethyl­ammonium 2-[(2-oxo-2*H*-chromen-7-yl)­oxy]acetate

**DOI:** 10.1107/S1600536811012761

**Published:** 2011-04-13

**Authors:** Feng-Xia Dong

**Affiliations:** a State Key Laboratory of Supramolecular Structure and Materials, College of Chemistry, Jilin University, Changchun 130012, People’s Republic of China

## Abstract

In the title salt, C_2_H_8_N^+^·C_11_H_7_O_5_^−^, the acetate group is twisted out of the plane of the coumarin ring system with a C—O—C—C torsion angle of 76.3 (2)°. In the crystal, N—H⋯O hydrogen bonds link the cations and anions into chains propagating in [100].

## Related literature

For the synthesis, see Matsuda *et al.* (2000 ▶). 
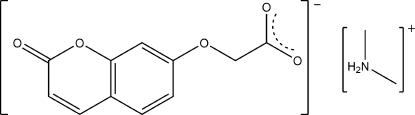


## Experimental

### 

#### Crystal data

C_2_H_8_N^+^·C_11_H_7_O_5_^−^
*M* *_r_* = 265.26
Triclinic, 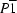

*a* = 6.714 (5) Å
*b* = 8.146 (7) Å
*c* = 12.767 (12) Å
α = 83.33 (4)°
β = 79.16 (3)°
γ = 67.78 (3)°
*V* = 634.1 (9) Å^3^
*Z* = 2
Mo *K*α radiation
μ = 0.11 mm^−1^
*T* = 293 K
0.44 × 0.22 × 0.14 mm

#### Data collection

Rigaku R-AXIS RAPID diffractometer
Absorption correction: multi-scan (*ABSCOR*; Higashi, 1995 ▶) *T*_min_ = 0.955, *T*_max_ = 0.986
6310 measured reflections
2881 independent reflections
1878 reflections with *I* > 2σ(*I*)
*R*_int_ = 0.026

#### Refinement

*R*[*F* ^2^ > 2σ(*F* ^2^)] = 0.041
*wR*(*F* ^2^) = 0.131
*S* = 0.93
2881 reflections
182 parameters
2 restraints
H atoms treated by a mixture of independent and constrained refinement
Δρ_max_ = 0.17 e Å^−3^
Δρ_min_ = −0.16 e Å^−3^

### 

Data collection: *RAPID-AUTO* (Rigaku, 1998 ▶); cell refinement: *RAPID-AUTO*; data reduction: *CrystalStructure* (Rigaku/MSC, 2002) ▶; program(s) used to solve structure: *SHELXS97* (Sheldrick, 2008 ▶); program(s) used to refine structure: *SHELXL97* (Sheldrick, 2008 ▶); molecular graphics: *SHELXTL* (Sheldrick, 2008 ▶); software used to prepare material for publication: *SHELXL97*.

## Supplementary Material

Crystal structure: contains datablocks I, global. DOI: 10.1107/S1600536811012761/ng5144sup1.cif

Structure factors: contains datablocks I. DOI: 10.1107/S1600536811012761/ng5144Isup2.hkl

Additional supplementary materials:  crystallographic information; 3D view; checkCIF report

## Figures and Tables

**Table 1:** Hydrogen-bond geometry (Å, °)

*D*—H⋯*A*	*D*—H	H⋯*A*	*D*⋯*A*	*D*—H⋯*A*
N1—H1*A*⋯O4	0.90 (1)	1.92 (1)	2.799 (2)	166 (2)
N1—H1*B*⋯O5^i^	0.90 (1)	1.86 (1)	2.729 (3)	160 (2)

Symmetry code: (i) 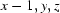.

**Fig. 1.**
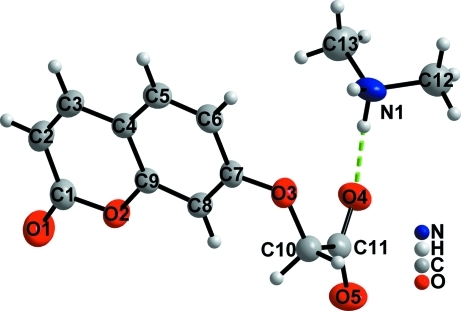
Molecular structure of the title compound, showing displacement ellipsoids at the 50% probability level for non-H atoms. Dashed lines indicate the hydrogen-bonding interactions.

**Fig. 2.**
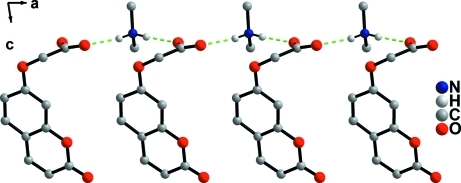
A partial packing view, showing the hydrogen-bonding chain structure. Dashed lines indicate the hydrogen bonds, no involving H atoms have been omitted for clarity.

## supplementary crystallographic information

### Comment

Coumarin derivatives have been widely studied due to the applications in medicine and optical materials. In this paper, we report the synthesis and crystal structure of the title compound, which is a type of carboxyl modified coumarin derivative.

In the title compound, the acetate group twist outside the plane of coumarin group with a C7—O3—C10—C11 torsion angle of 76.3 (2). The hydrogen atom of carboxyl transfer to the dimethylamine molecule forming N—H···O hydrogen bonding interaction (Figure 1).

In the crystal structure of the title compound, the N—H···O hydrogen bonds bewteen coumarin anions and dimethylammonium cations link them to form a chain structure (Figure 2, Table 1).

### Experimental

A mixture of 7-hydroxycoumarin (0.16 g, 1.0 mmol), potassium carbonate (0.20 g, 1.4 mmol), ethyl bromoacetate (0.20 g, 1.2 mmol), and dry acetone (30 ml) was refluxed for 4 h while stirring in a N_2_ atmosphere. After removal of salt by filtration, the resulting ester was recrystallized from ethanol. After then, the carboxylic acid derivatives was obtained through refluxing in sodium hydroxide solution and protonized with HCl. Mix the obtained carboxylic acid derivatives with dimethylamine with molar ratio of 1:1 in methanol, needle-like crystals of title compound were obtained after several days.

### Refinement

The reflection data (2 3 2) had been omit in the refinement. H atoms bound to C atoms were placed in calculated positions and treated as riding on their parent atoms, with C—H = 0.93 Å (aromatic C), C—H = 0.97Å (methylene C), and with *U*_iso_(H) = 1.2Ueq(C) or C—H = 0.96 Å (methly C) and with *U*_iso_(H) = 1.5Ueq(C). The N-bound H atoms were initially located in a difference Fourier map and they were refined with N—H=0.90 Å.

### Crystal data

**Table:**

C_2_H_8_N^+^·C_11_H_7_O_5_^−^	*Z* = 2
*M**_r_* = 265.26	*F*(000) = 280
Triclinic, *P*1	*D*_x_ = 1.389 Mg m^−^^3^
Hall symbol: -P 1	Mo *K*α radiation, λ = 0.71073 Å
*a* = 6.714 (5) Å	Cell parameters from 4269 reflections
*b* = 8.146 (7) Å	θ = 3.1–27.5°
*c* = 12.767 (12) Å	µ = 0.11 mm^−^^1^
α = 83.33 (4)°	*T* = 293 K
β = 79.16 (3)°	Block, colorless
γ = 67.78 (3)°	0.44 × 0.22 × 0.14 mm
*V* = 634.1 (9) Å^3^	

### Data collection

**Table:**

Rigaku R-AXIS RAPID diffractometer	2881 independent reflections
Radiation source: fine-focus sealed tube	1878 reflections with *I* > 2σ(*I*)
graphite	*R*_int_ = 0.026
ω scans	θ_max_ = 27.5°, θ_min_ = 3.1°
Absorption correction: multi-scan (*ABSCOR*; Higashi, 1995)	*h* = −8→8
*T*_min_ = 0.955, *T*_max_ = 0.986	*k* = −10→10
6310 measured reflections	*l* = −16→16

### Refinement

**Table:**

Refinement on *F*^2^	Primary atom site location: structure-invariant direct methods
Least-squares matrix: full	Secondary atom site location: difference Fourier map
*R*[*F*^2^ > 2σ(*F*^2^)] = 0.041	Hydrogen site location: inferred from neighbouring sites
*wR*(*F*^2^) = 0.131	H atoms treated by a mixture of independent and constrained refinement
*S* = 0.93	*w* = 1/[σ^2^(*F*_o_^2^) + (0.0824*P*)^2^ + 0.0078*P*] where *P* = (*F*_o_^2^ + 2*F*_c_^2^)/3
2881 reflections	(Δ/σ)_max_ < 0.001
182 parameters	Δρ_max_ = 0.17 e Å^−^^3^
2 restraints	Δρ_min_ = −0.16 e Å^−^^3^

### Special details

**Table:**

Geometry. All e.s.d.'s (except the e.s.d. in the dihedral angle between two l.s. planes) are estimated using the full covariance matrix. The cell e.s.d.'s are taken into account individually in the estimation of e.s.d.'s in distances, angles and torsion angles; correlations between e.s.d.'s in cell parameters are only used when they are defined by crystal symmetry. An approximate (isotropic) treatment of cell e.s.d.'s is used for estimating e.s.d.'s involving l.s. planes.
Refinement. Refinement of *F*^2^ against ALL reflections. The weighted *R*-factor *wR* and goodness of fit *S* are based on *F*^2^, conventional *R*-factors *R* are based on *F*, with *F* set to zero for negative *F*^2^. The threshold expression of *F*^2^ > σ(*F*^2^) is used only for calculating *R*-factors(gt) *etc*. and is not relevant to the choice of reflections for refinement. *R*-factors based on *F*^2^ are statistically about twice as large as those based on *F*, and *R*- factors based on ALL data will be even larger.

### Fractional atomic coordinates and isotropic or equivalent isotropic displacement parameters (Å^2^)

**Table:**

	*x*	*y*	*z*	*U*_iso_*/*U*_eq_	
N1	0.1702 (2)	0.71671 (17)	0.56112 (12)	0.0410 (3)	
C1	0.4408 (3)	0.6603 (2)	1.12637 (14)	0.0474 (4)	
C2	0.2575 (3)	0.6054 (3)	1.16133 (14)	0.0509 (4)	
H2	0.2490	0.5412	1.2261	0.061*	
C3	0.0990 (3)	0.6456 (2)	1.10186 (14)	0.0492 (4)	
H3	−0.0200	0.6120	1.1270	0.059*	
C4	0.1103 (2)	0.7390 (2)	1.00064 (12)	0.0390 (4)	
C5	−0.0479 (2)	0.7886 (2)	0.93318 (14)	0.0469 (4)	
H5	−0.1734	0.7628	0.9553	0.056*	
C6	−0.0222 (2)	0.8737 (2)	0.83609 (13)	0.0441 (4)	
H6	−0.1294	0.9053	0.7929	0.053*	
C7	0.1668 (2)	0.9134 (2)	0.80145 (12)	0.0352 (3)	
C8	0.3255 (2)	0.8682 (2)	0.86578 (12)	0.0377 (4)	
H8	0.4510	0.8942	0.8435	0.045*	
C9	0.2930 (2)	0.7836 (2)	0.96401 (12)	0.0366 (4)	
C10	0.3639 (2)	1.0429 (2)	0.66355 (12)	0.0366 (4)	
H10A	0.3951	1.0951	0.7201	0.044*	
H10B	0.3299	1.1324	0.6057	0.044*	
C11	0.5675 (2)	0.8869 (2)	0.62315 (12)	0.0340 (3)	
C12	0.1952 (3)	0.7031 (3)	0.44443 (15)	0.0557 (5)	
H12A	0.0662	0.6949	0.4271	0.084*	
H12B	0.3187	0.5991	0.4223	0.084*	
H12C	0.2168	0.8065	0.4081	0.084*	
C13	0.1659 (3)	0.5537 (3)	0.62202 (18)	0.0646 (5)	
H13A	0.2810	0.4530	0.5894	0.097*	
H13B	0.0281	0.5430	0.6225	0.097*	
H13C	0.1856	0.5584	0.6940	0.097*	
O1	0.5856 (2)	0.6407 (2)	1.17600 (11)	0.0675 (4)	
O2	0.45322 (16)	0.74415 (16)	1.02652 (9)	0.0459 (3)	
O3	0.17667 (14)	0.99754 (15)	0.70317 (8)	0.0410 (3)	
O4	0.54894 (16)	0.75011 (15)	0.60073 (10)	0.0472 (3)	
O5	0.73879 (16)	0.91839 (17)	0.61151 (11)	0.0571 (4)	
H1B	0.040 (2)	0.802 (2)	0.5816 (16)	0.067 (6)*	
H1A	0.279 (2)	0.747 (3)	0.5738 (16)	0.068 (6)*	

### Atomic displacement parameters (Å^2^)

**Table:**

	*U*^11^	*U*^22^	*U*^33^	*U*^12^	*U*^13^	*U*^23^
N1	0.0340 (6)	0.0366 (7)	0.0567 (9)	−0.0137 (5)	−0.0116 (6)	−0.0086 (6)
C1	0.0462 (9)	0.0598 (11)	0.0368 (9)	−0.0195 (8)	−0.0072 (7)	−0.0023 (8)
C2	0.0554 (10)	0.0640 (11)	0.0366 (9)	−0.0294 (9)	−0.0012 (8)	0.0013 (8)
C3	0.0459 (9)	0.0642 (11)	0.0437 (10)	−0.0311 (8)	0.0046 (7)	−0.0071 (9)
C4	0.0322 (7)	0.0512 (9)	0.0375 (9)	−0.0207 (6)	0.0018 (6)	−0.0102 (7)
C5	0.0318 (7)	0.0694 (11)	0.0474 (10)	−0.0274 (7)	−0.0002 (7)	−0.0124 (9)
C6	0.0289 (7)	0.0650 (11)	0.0413 (9)	−0.0179 (7)	−0.0062 (6)	−0.0105 (8)
C7	0.0275 (7)	0.0447 (8)	0.0315 (8)	−0.0107 (6)	−0.0008 (6)	−0.0087 (7)
C8	0.0267 (7)	0.0534 (9)	0.0358 (8)	−0.0189 (6)	−0.0009 (6)	−0.0046 (7)
C9	0.0280 (7)	0.0492 (9)	0.0345 (8)	−0.0152 (6)	−0.0035 (6)	−0.0080 (7)
C10	0.0345 (7)	0.0388 (8)	0.0361 (8)	−0.0131 (6)	−0.0043 (6)	−0.0026 (7)
C11	0.0302 (7)	0.0415 (8)	0.0315 (8)	−0.0125 (6)	−0.0090 (6)	−0.0007 (6)
C12	0.0580 (10)	0.0542 (10)	0.0547 (11)	−0.0179 (8)	−0.0144 (9)	−0.0018 (9)
C13	0.0706 (12)	0.0565 (11)	0.0663 (13)	−0.0243 (10)	−0.0181 (10)	0.0147 (10)
O1	0.0595 (8)	0.1038 (11)	0.0491 (8)	−0.0383 (8)	−0.0243 (6)	0.0135 (8)
O2	0.0351 (5)	0.0709 (8)	0.0374 (6)	−0.0263 (5)	−0.0095 (5)	0.0047 (6)
O3	0.0282 (5)	0.0550 (7)	0.0365 (6)	−0.0115 (4)	−0.0052 (4)	−0.0019 (5)
O4	0.0392 (6)	0.0417 (6)	0.0615 (8)	−0.0118 (5)	−0.0088 (5)	−0.0145 (6)
O5	0.0301 (5)	0.0638 (8)	0.0809 (10)	−0.0189 (5)	−0.0076 (6)	−0.0134 (7)

### Geometric parameters (Å, °)

**Table:**

N1—C13	1.466 (3)	C7—O3	1.361 (2)
N1—C12	1.480 (3)	C7—C8	1.381 (2)
N1—H1B	0.904 (9)	C8—C9	1.381 (2)
N1—H1A	0.899 (9)	C8—H8	0.9300
C1—O1	1.209 (2)	C9—O2	1.378 (2)
C1—O2	1.378 (2)	C10—O3	1.4287 (19)
C1—C2	1.441 (3)	C10—C11	1.524 (2)
C2—C3	1.341 (3)	C10—H10A	0.9700
C2—H2	0.9300	C10—H10B	0.9700
C3—C4	1.426 (3)	C11—O4	1.238 (2)
C3—H3	0.9300	C11—O5	1.2490 (19)
C4—C9	1.392 (2)	C12—H12A	0.9600
C4—C5	1.404 (2)	C12—H12B	0.9600
C5—C6	1.362 (3)	C12—H12C	0.9600
C5—H5	0.9300	C13—H13A	0.9600
C6—C7	1.406 (2)	C13—H13B	0.9600
C6—H6	0.9300	C13—H13C	0.9600
			
C13—N1—C12	112.95 (16)	C9—C8—H8	120.9
C13—N1—H1B	106.2 (13)	C7—C8—H8	120.9
C12—N1—H1B	107.5 (14)	O2—C9—C8	116.35 (13)
C13—N1—H1A	111.7 (14)	O2—C9—C4	120.33 (15)
C12—N1—H1A	107.7 (13)	C8—C9—C4	123.32 (14)
H1B—N1—H1A	110.8 (19)	O3—C10—C11	114.33 (13)
O1—C1—O2	116.32 (16)	O3—C10—H10A	108.7
O1—C1—C2	126.56 (18)	C11—C10—H10A	108.7
O2—C1—C2	117.12 (15)	O3—C10—H10B	108.7
C3—C2—C1	120.98 (18)	C11—C10—H10B	108.7
C3—C2—H2	119.5	H10A—C10—H10B	107.6
C1—C2—H2	119.5	O4—C11—O5	127.04 (13)
C2—C3—C4	120.98 (15)	O4—C11—C10	119.35 (13)
C2—C3—H3	119.5	O5—C11—C10	113.50 (14)
C4—C3—H3	119.5	N1—C12—H12A	109.5
C9—C4—C5	116.59 (16)	N1—C12—H12B	109.5
C9—C4—C3	118.16 (15)	H12A—C12—H12B	109.5
C5—C4—C3	125.24 (14)	N1—C12—H12C	109.5
C6—C5—C4	121.69 (14)	H12A—C12—H12C	109.5
C6—C5—H5	119.2	H12B—C12—H12C	109.5
C4—C5—H5	119.2	N1—C13—H13A	109.5
C5—C6—C7	119.80 (14)	N1—C13—H13B	109.5
C5—C6—H6	120.1	H13A—C13—H13B	109.5
C7—C6—H6	120.1	N1—C13—H13C	109.5
O3—C7—C8	124.62 (13)	H13A—C13—H13C	109.5
O3—C7—C6	115.05 (13)	H13B—C13—H13C	109.5
C8—C7—C6	120.33 (15)	C9—O2—C1	122.27 (13)
C9—C8—C7	118.24 (13)	C7—O3—C10	117.87 (11)
			
C7—O3—C10—C11	76.32 (16)		

### Hydrogen-bond geometry (Å, °)

**Table:**

*D*—H···*A*	*D*—H	H···*A*	*D*···*A*	*D*—H···*A*
N1—H1A···O4	0.90 (1)	1.92 (1)	2.799 (2)	166.(2)
N1—H1B···O5^i^	0.90 (1)	1.86 (1)	2.729 (3)	160.(2)

Symmetry codes: (i) *x*−1, *y*, *z*.
